# Carbon Dioxide Insufflation in Colonoscopy Is Safe: A Prospective Trial of 347 Patients

**DOI:** 10.1155/2012/692532

**Published:** 2012-09-29

**Authors:** M. Geyer, U. Guller, Ch. Beglinger

**Affiliations:** ^1^Gastroenterologie Wettingen, Rosengartenstrasse 2, 5430 Wettingen, Switzerland; ^2^Department of Oncology, Kantonsspital, 9007 St. Gallen, Switzerland; ^3^Department of Visceral Surgery and Medicine, University Hospital Bern, 3010 Bern, Switzerland; ^4^Department of Gastroenterology, University Hospital, 4031 Basel, Switzerland

## Abstract

Available evidence suggests that the use of CO_2_ insufflation in endoscopy is more comfortable for the patient. The safety of CO_2_ use in colonoscopy remains contentious, particularly in sedated patients. The objective of the present prospective trial was to assess the safety of CO_2_ colonoscopies. *Methods*. 109 patients from our previous randomized CO_2_ colonoscopy study and an additional 238 subsequent consecutive unselected patients who had a routine colonoscopy performed in a private practice were enrolled from April 2008 through September 2008. All but 2 patients were sedated. All patients were routinely monitored with transcutaneous CO_2_ measurement. Volumes of CO_2_ administered were correlated with capnographic measurements from transcutaneous monitoring. *Results*. Of the 347 patients examined, 57% were women; mean (SD) age of participants was of 60.2 years (12.8). Mean propofol dosage was 136 mg (64 mg). Mean CO_2_ values were 34.7 mm Hg (5.3) at baseline, 38.9 mm Hg (5.5) upon reaching the ileum, and 36.9 mm Hg (5.0) at examination's end. Mean maximum increase of CO_2_ was 4.5 mm Hg (3.6). No correlation was observed between volume of CO_2_ administered and increase in level of CO_2_ (correlation coefficient: 0.01; *P* value: 0.84). No complications were observed. *Conclusions*. The present prospective study, which was based on one of the largest sedated patient sample reported to date in this setting, provides compelling evidence that CO_2_ insufflation in colonoscopy is safe and unassociated with relevant increases in transcutaneously measured levels of CO_2_.

## 1. Background 

For almost 2 decades, CO_2_ insufflation with carbon dioxide (CO_2_) has been widely used in laparoscopic surgery. In contrast, insufflation with room air has remained the standard of care in the vast majority of endoscopy centers in both Europe and North America. Unfortunately, many patients still experience pain and discomfort after colonoscopy with room air insufflation. Studies [1–5] and one review [6] indicate that insufflation with CO_2_ can reduce periprocedural pain in different endoscopic settings (e.g., endoscopic retrograde cholangio-pancreatography [7], balloon enteroscopy [8], and endoscopic submucosal dissection [9]). However, in the setting of colonoscopy, current scientific data with regard to the safety of CO_2_ insufflation are limited. Small patient populations have characterized most studies, including those with sedated patients, and many physicians remain concerned that CO_2_ insufflation might lead to CO_2_ retention. In a previous randomized, controlled, double-blinded trial, we randomly allocated 219 patients to colonoscopy with CO_2_ versus room air, patients in the CO_2_ group experienced significantly less pain and bloating and a higher overall satisfaction score [10]. However, this study was not designed to definitively demonstrate the safety of CO_2_ insufflation in colonoscopy. In the present study, we sought to prospectively assess the safety of CO_2_ colonoscopy in a larger sample of patients. 

## 2. Patients and Methods 

This trial included all 109 patients from our previous CO_2_ colonoscopy study [10] plus 238 subsequent, consecutive, nonselected, patients who had a routine colonoscopy performed in a private Swiss gastroenterology practice and were enrolled from April 2008 through September 2008. All patients who were deemed medically fit for an ambulatory colonoscopy were included in this study. In order to maximize the generalizability (external validity) of our study, we did not initially apply exclusion criteria, although one exclusion criterion emerged (due to a small earlap, CO_2_ measurement was not possible in 4 patients, which were excluded). All colonoscopies were done by the first author (M. G.) and carried out with standard Pentax endoscopes (EC-3885K and EC-380FKp) (Pentax Medical, Pentax of America, Inc., Montvale, NJ, USA) with an EPK 1000 processor. For CO_2_ insufflation, the CO_2_-Efficent Insufflator device (EZEM Company, Westbury, NY, USA) was used. The insufflator was connected to a 10-litre CO_2_ bottle and supplied over a tubing set connected with a branch connection to the water bottle tube, which was itself directly connected to the endoscope. The flow rate (basal flow rate 0.5 L/min, increasing to 3 L/min maximum) can be controlled on demand over the standard air valve. Oxygen was delivered to patients if the saturation dropped below 90% at a flow rate of 4 liters per minute.

All but 2 patients were sedated with propofol using standard procedures previously described [11]. The total dose of propofol was registered for later analysis. A level of conscious sedation (“moderate sedation”) was targeted. All patients were routinely monitored with transcutaneous CO_2_ measurement on the ear, with the exception of those patients who were excluded due to insufficient earlap (*n* = 4). CO_2_ measurement was performed with the SenTec capnograph (SenTec AG, 4106 Therwil, Switzerland) as described by Heuss et al. [12]. Our measurement technique followed that recommended by SenTec; internal validation was not performed as the system has been previously extensively validated [12–14]. Sensors were calibrated according to mandatory procedure and placed on the earlap after cleaning with 70% isopropyl alcohol solution and application of contact liquid on the sensor membrane.

In previous validation studies, correlation of partial pressure of CO_2_ (paCO_2_) and transcutaneous CO_2_ measurement was as follows: *r* = 0.92; intraclass correlation coefficient [ICC] = 0.92; for arterial saturation of oxygen (SaO_2_/SpO_2_), *r* = 0.74; ICC = 0.73 [14]. Data for continuous transcutaneous CO_2_ measurements were analyzed, as were baseline characteristics including age, propofol use, and CO_2_ volume administered. To ensure validity of our measurements, we analyzed and compared the 238 consecutive patients separately, with the 109 patients derived from our earlier CO_2_ study using an unpaired *t*-test. Additionally the correlation of the insufflated amount of CO_2_ with a possible increase of transcutaneously measured CO_2_ was assessed using Spearman correlations. Statistical analyses were performed using SPSS, v. 11.0 (IBM Corp., Armonk, NY, USA).

## 3. Results 

**Figure 1:**
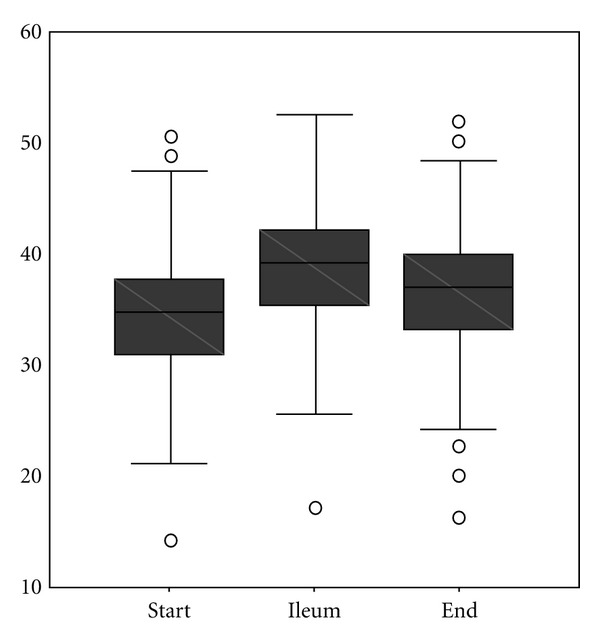
CO_2_ values over time (mm Hg).

**Figure 2:**
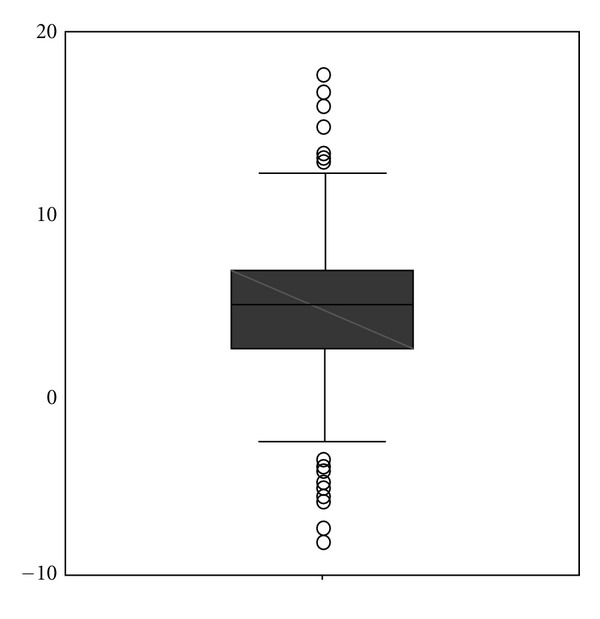
Increase in CO_2_ (mm Hg).

The first sample of 109 patients who received CO_2_ insufflation for colonoscopy was drawn from our previous study, which was conducted from April to June of 2008. All subsequent patients referred for colonoscopy at this single practice were routinely examined with CO_2_, with the result that from June 2008 through September 2008, an additional 238 patients (second sample) were examined using CO_2_ insufflation, for a final study population of 347. Of the 347 patients examined, 198 (57%) were female; the mean (SD) age was 60 years (12.8). Mean (SD) propofol dosage was 136 mg (64) (Table 1); mean (SD) duration of endoscopy procedures was 24 minutes (7.5). All but 2 patients in the second sample were sedated. Mean (SD) value for baseline CO_2_ measurement was 34.7 mm Hg (5). Mean (SD) pressure of CO_2_ recorded reaching the ileum was 38.9 mm Hg (5) and 36.9 mm Hg (5) at the end of the examination (Figure 1). Mean maximum increase of CO_2_ pressure was 4.4 mm Hg (Figure 2). 

Four patients in the second sample experienced transient pCO_2_ values in excess of 50 mm Hg (three when reaching the ileum and one at the end of the examination), but these patients showed no signs of respiratory distress or O_2_ desaturation and O_2_ substitution was not deemed necessary. These four patients did not receive a higher propofol dosage or volume of CO_2_ insufflated. 

**Figure 3:**
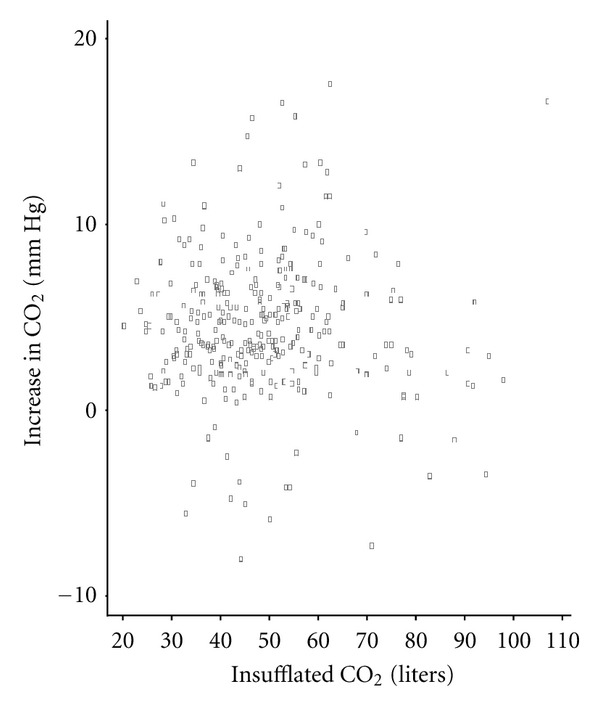
Correlation of CO_2_ change and insufflated CO_2_ volume.

Among the total study sample, no correlation was observed between volume of CO_2_ administered and an increase of CO_2_ (correlation coefficient: 0.01; *P*-value: 0.84) (Figure 3). Parameters did not significantly differ between the first and second samples. None of the patients required ventilation or mechanical airway support, and no other complications (bleeding, perforation, loss of consciousness, hospital admission) were observed.

## 4. Discussion

The present prospective study, which was based on one of the largest sample of sedated patients to date, provides compelling evidence that CO_2_ insufflation colonoscopy is safe and is not associated with an increase in transcutaneously measured CO_2_ or with adverse respiratory effects. Our findings add to mounting scientific evidence demonstrating the safety and superiority of CO_2_ colonoscopy over room air [6, 10]. Given these findings, we believe that CO_2_ colonoscopy should be considered the procedure of choice. 

Although CO_2_ insufflation has been used for different endoscopic procedures [1–9] and was found to be associated with less postprocedural pain and bloating [10, 15], most endoscopy units continue to use room air for screening colonoscopies and are unaware of the benefits of this relatively new technique [16]. 

In our first double-blinded randomized study—hitherto the largest reported sample of sedated and CO_2_ monitored patients—239 patients were randomly allocated in a 1 : 1 ratio to colonoscopy with CO_2_ versus room air. This study, which represented the first examination of sedated, unselected, and consecutive patients in this setting, yielded encouraging results: no difference in transcutaneous CO_2_ values was observed with continuous capnography between groups, and there was no evidence of advanced hypercapnia as a possible problem with this technique [10]. However, we felt that this initial experience from our group needed to be confirmed in a study with larger sample size. By incorporating patients randomized to CO_2_ insufflation in that previous study and an additional 238 consecutive patients, we achieved an even larger patient population, providing compelling evidence for the safety of CO_2_ insufflation in sedated and monitored patients undergoing colonoscopy. Only 4 patients experienced a partial pressure of CO_2_ in excess of 50 mm Hg (max. paCO_2_ 52.8 mm Hg). 

Our study shows that sedated patients had a small increase in CO_2_ during colonoscopy until the terminal ileum was reached, after which CO_2_ levels fell. We speculate from these observations that sedation per se, but not CO_2_ insufflation in particular, appears to be the primary cause of the observed CO_2_ increase during colonoscopy. 

Many patients with severe chronic obstructive pulmonary disease (COPD) experience CO_2_ retention, an inherent phenomenon of this disease. Until now, no studies have been performed to assess whether CO_2_ is also safe to use in this high-risk population. But although COPD patients included in our study suffered no relevant adverse outcomes, they constituted only a small percentage of the study population and larger studies of CO_2_ insufflation in patients with COPD should be performed to confirm its safety in this subset of patients.

A particularly noteworthy finding of this study is the lack of correlation between volume of insufflated CO_2_ and the increase of CO_2_ as measured by transcutaneous monitoring. Because CO_2_ is very quickly exhaled, paCO_2_ never reached clinically toxic value. It might be influenced more by individual factors not yet fully understood than by the amount of insufflated CO_2_. 

Because CO_2_ is absorbed into the bloodstream 150 times faster than room air and also exhaled with an elimination of 0.2 L per minute [17], higher volumes of gas (e.g., 51 L [±14 L]) are usually needed when using CO_2_ for colonoscopy insufflation, compared with room air [18]. Some physicians who are already using CO_2_ insufflation for colonoscopy believe that briefly switching from CO_2_ to room air to obtain a faster and better colonic distension can be useful in certain circumstances. This may be due to the fact that the flow rates of all commercial available CO_2_ insufflators are still below the usual rates achieved with air insufflators. 

From a practical point of view, there are no clinically relevant problems in handling CO_2_ insufflation with modern insufflators; further, we believe that no safety concerns remain in patients devoid of COPD. 

## 5. Conclusion 

The present prospective study, which was based on one of the largest sedated patient sample reported to date, provides compelling evidence that colonoscopy with CO_2_ insufflation is safe in patients devoid of COPD. Neither a relevant increase in transcutaneously measured CO_2_ nor adverse respiratory effects were detected. 

## Figures and Tables

**Table 1:** Results (mean ± SD)^1^.

	1st sample (*n* = 109)	2nd sample (*n* = 238)	All (*n* = 347)
Sex, female	62%	55%	57%
Propofol (mg)	134 ± 56	137 ± 68	136 ± 64
CO_2_ start (mm Hg)	33.4 ± 4.7	35.4 ± 5.4	34.7 ± 5.3
CO_2_ ileum (mm Hg)	37.3 ± 5.2	39.6 ± 5.5	38.9 ± 5.5
CO_2_ end (mm Hg)	35.2 ± 4.3	37.7 ± 5.1	36.9 ± 5.0
Amount CO_2_ (L)	51 ± 14	47 ± 15	48 ± 15

^1^ All *P* values > 0.05.
